# Common pitfalls, and how to avoid them, in child and adolescent psychopharmacology: Part II

**DOI:** 10.1177/02698811241239596

**Published:** 2024-03-17

**Authors:** Samuele Cortese, Frank MC Besag, Bruce Clark, Chris Hollis, Joe Kilgariff, Carmen Moreno, Dasha Nicholls, Paul Wilkinson, Marc Woodbury-Smith, Aditya Sharma

**Affiliations:** 1 Centre for Innovation in Mental Health, School of Psychology, Faculty of Environmental and Life Sciences, University of Southampton, Southampton, UK; 2 Solent NHS Trust, Southampton, UK; 3 Hassenfeld Children’s Hospital at NYU Langone, New York University Child Study Center, New York City, New York, USA; 4 Department of Precision and Regenerative Medicine-Jonic Area (DiMePRe-J), University of Bari “Aldo Moro”, Bari, Italy; 5 UCL School of Pharmacy, London, UK; 6 East London Foundation NHS Trust, Bedfordshire, UK; 7 National Specialist Clinic for Young People with OCD, BDD and Related Disorders, South London and Maudsley NHS Foundation Trust, London, UK; 8 Department of Child and Adolescent Psychiatry, Nottinghamshire Healthcare NHS Foundation Trust, Queen’s Medical Centre, Nottingham, UK; 9 National Institute of Mental Health (NIHR) MindTech Medtech Co-operative, Institute of Mental Health, Nottingham, UK; 10 NIHR Nottingham Biomedical Research Centre, Mental Health & Technology Theme, Institute of Mental Health, Nottingham, UK; 11 Department of Child and Adolescent Psychiatry, Institute of Psychiatry and Mental Health, Hospital General Universitario Gregorio Marañón, IiSGM, CIBERSAM, ISCIII, School of Medicine, Universidad Complutense, Madrid, Spain; 12 Division of Psychiatry, Imperial College London, London, UK; 13 NIHR ARC Northwest London, London, UK; 14 School of Clinical Medicine, University of Cambridge, Cambridge, Cambridgeshire, UK; 15 Cambridgeshire and Peterborough NHS Foundation Trust, Cambridge, UK; 16 Biosciences Institute, Newcastle University, Newcastle upon Tyne, UK; 17 Academic Psychiatry, Translational and Clinical Research Institute, Newcastle University, Newcastle upon Tyne, UK; 18 Specialist Adolescent Mood Disorders Service (SAMS), Cumbria, Northumberland, Tyne and Wear NHS Foundation Trust, Newcastle upon Tyne, UK

**Keywords:** Psychopharmacology, children, adolescents, young people, pitfalls, expert opinion

## Abstract

As Faculty of the British Association for Psychopharmacology course on child and adolescent psychopharmacology, we present here what we deem are the most common pitfalls, and how to avoid them, in child and adolescent psychopharmacology. In this paper, we specifically addressed common pitfalls in the pharmacological treatment of autism and intellectual disability, eating disorders, neuropsychiatric correlates of epilepsy, and psychosis. Pitfalls in relation to the treatment of other disorders are addressed in a separate paper (Part I).

## Introduction

As Faculty of the British Association for Psychopharmacology (BAP) course on child and adolescent psychopharmacology, we previously published a paper (Cortese et al., 2023) reporting the most common questions we have been asked in recent editions of the course, alongside evidence-based and/or expert-informed answers. Here, based on our experience during the course, we have selected what we deem are the most common pitfalls, and how to avoid them, in child and adolescent psychopharmacology, focusing on autism and intellectual disability, eating disorders, neuropsychiatric correlates of epilepsy, and psychosis. We have grouped the pitfalls by disorder to which they refer, in alphabetical order. Pitfalls in relation to the treatment of other disorders (attention-deficit/hyperactivity disorder (ADHD), anxiety, bipolar disorder, depression, obsessive-compulsive disorder and related disorders, and tic disorder) are addressed in a separate paper (Part I).

## Autism and intellectual disability

### Increasing the dose of medication in the absence of a complete diagnostic formulation

Among young people with autism and intellectual disability (ID), challenging behaviour represents a common reason for referral to specialist mental health services. In a multidisciplinary setting, non-pharmacological options are always explored first. Typically, behavioural assessment, alongside insight from professionals such as occupational therapists and communication therapists, results in a diagnostic formulation with specific treatment recommendations. In some instances, a mental illness may also be suspected, which, if diagnosed, warrants pharmacological treatment according to good practice guidelines and existing treatment protocols (Deb et al., 2023; Sheehan et al., 2015). However, even in the absence of a mental illness, there is sometimes an indication for pharmacological intervention, for example, if the severity of challenging behaviour does not allow behavioural interventions to proceed.

Often, certain antipsychotics, such as risperidone (D2, 5-HT2 and NE alpha 2 receptor antagonist) or aripiprazole (D2 and 5-HT1A receptor partial agonist), can positively impact and reduce behaviour even in the absence of an underlying mental illness (Groves et al., 2023). However, when these medications are not helping, it is quite usual for requests to be made for an increase in medication dose. Before doing so, the reason for the lack of efficacy should be explored. For example, sometimes medications help at first but then their effect seems to ‘wear off’. Some families may even report an initial positive impact even before an effect is biologically plausible. This is presumably due to a placebo effect. As such, increasing the medication in such instances would seem inappropriate. On the other hand, increasing the dose of medication too rapidly may simply introduce the unwanted effect of sedation, which itself may be interpreted as evidence of efficacy. Sedation will clearly result in the diminution of challenging behaviour but at a cost of reduced quality of life. Indeed, in all instances of medication use, it is important that realistic target outcomes are clearly defined and agreed upon. Ultimately, if the ‘function’ of the behaviour is poorly understood, it is unlikely that pharmacology will help, other than through this sedative effect. Because of these various factors, changes to medication should only take place slowly and in full discussion with the multidisciplinary team, to understand the underlying reasons for the behaviour.

### Misdiagnosing challenging behaviour as anxiety

The term *anxiety* is used very frequently among carers of young people with autism and ID. While it is true that anxiety is common in this population (Totsika et al., 2022), the term should not be taken at face value when collecting clinical information. Indeed, the term is oftentimes used by carers and others synonymously with challenging behaviour itself; as such, it may offer very little insight into underlying psychopathology and its aetiology. Clinicians, on the other hand, should only ever use the term anxiety according to its nosological criteria as set out in the Diagnostic and Statistical Manual of mental Disorders (DSM) or in the International Classification of Diseases (ICD) . Importantly, the criteria for anxiety disorders are identical for people with ID and their peers. In short, standard ICD or DSM criteria and thresholds apply, although greater emphasis may be placed on behavioural characteristics. Ultimately, it is the role of the health professionals, not carers, educators or support staff, to make judgement on diagnostic entities such as anxiety, and to use diagnostic terms appropriately.

### Deciding on the threshold for an Obsessive Compulsive Disorder (OCD) diagnosis in ID

Ritualistic patterns of behaviour are very common in ID, particularly so among those who have an additional diagnosis of autism. Ritualistic behaviours can often be understood as consistent with the individual’s developmental rather than chronological age. In other cases, ritualistic behaviours are ‘comforting’ or carried out to reduce stress during difficult times. In such instances, it is not the behaviour itself that is causing the stress. Indeed, the behaviour is likely a positive, egosyntonic experience.

On the other hand, some behaviours are clearly associated with stress. This can often be evident by simple observation. Whether or not these behaviours are causally associated with underlying cognitions (obsessive thoughts), which may be difficult for the individual to verbalise, distress is evident and so it is reasonable to conclude that they are egodystonic. In this latter situation, a diagnosis of OCD may be considered, and pharmacological and behavioural treatments may be initiated. It would certainly be unreasonable to eliminate rituals merely because they cause inconvenience to others if they do not impact the well-being and quality of life of the child.

### The importance of an ‘exit strategy’ when prescribing antipsychotics for challenging behaviours

Evidence on the use of low doses of antipsychotics for disruptive behaviours in children is limited to short-term studies (Rajkumar, 2022). Notably, as in individuals with disabilities, pharmacological treatments are often unnecessarily long, without discontinuation even after a persisting clinical improvement, ‘exit strategy’ (i.e. discussing the need to consider stopping a medication once the disruptive behaviour is stabilised) should always be planned with the patients and their caregivers.

## Eating disorders

### Prescribing unnecessarily

Comorbidity is the norm alongside eating disorder diagnoses. Evidence suggests comorbidity rates, principally depression, anxiety and OCD, for adolescent anorexia nervosa range from 27% to 58%, and for bulimia nervosa, principally depression, from 48% to 60% (Hambleton et al., 2022). The treatment of comorbidities is not always necessary since the treatment for eating disorders includes strategies that facilitate emotional communication and coping skills. Consequently, without the need for additional treatment, depression and anxiety often resolve following the treatment of the eating disorder (Lock and Nicholls, 2019). Evidence suggests that, in some adult cases, selective serotonin reuptake inhibitors (SSRIs) may be useful in relapse prevention where symptoms do not fully resolve (Kaye et al., 2001), although this has not been demonstrated in adolescents. The converse is not true – treatment for anxiety or depression is unlikely to improve eating disorder symptoms.

### Refusal and non-adherence

Antipsychotic medications, specifically olanzapine (D2, 5-HT2 receptor antagonist) and aripiprazole, are effective in a small number of trials, case series and case reports in adults and adolescents with anorexia nervosa (Himmerich et al., 2023). However, the reluctance of patients to take olanzapine (Attia et al., 2011) and low adherence and acceptability rates (Attia et al., 2019) negate much of the potential efficacy of these medications and may account for why large clinical trials have not been possible to conduct in adolescent samples (e.g. a recent multisite olanzapine feasibility study – Himmerich et al., 2023, personal communication). One possible reason for this lack of adherence is the focus on weight gain as the primary mechanism of action and the primary objective of olanzapine prescription. This is neither helpful nor, arguably, correct, although no mechanistic studies have been conducted as yet. Issues with accepting and tolerating weight gain as a desired outcome are central to the psychopathology of anorexia nervosa. Effective prescribing therefore requires reassurance that weight gain will not escalate over and above that expected for the standard treatment approach (typically eating disorders focussed family therapy) and that the purpose of the medication is to moderate emotional arousal associated with the first phases of treatment to manageable levels, without inducing somnolence. This is based on the sedative, anxiolytic and mood-regulating effects of antipsychotics, which may enable them to reduce obsessive ideas and anxiety, improving mood stability (Thorey et al., 2023).

### Underestimation of additional risks of prescribing

Another pitfall of note is an underestimation of the risks associated with prescribing across the eating disorder spectrum. Nutritional instability, independent of weight status, can increase the risk of adverse effects, many of which are documented in case reports (Himmerich et al., 2023). Examples include hyper- or hypoglycaemia with olanzapine or QTc prolongation with antipsychotics in general. Stimulant medications are potentially under-prescribed in children and young people with binge eating disorder. Indeed, practitioners should consider (a) the relationship between binge eating disorder and ADHD; (b) the neurobiological rationale; and (c) the current evidence, limited to adults, for stimulants as treatments for binge eating disorder. However, the possible benefits of stimulant medications must be balanced with risks such as the potential for medication misuse, adverse cardiovascular events, and reduction of appetite and pathological weight loss. Further research is warranted to assess the risks and benefits of the use of stimulants in youth with eating disorders (Keshen et al., 2022).

## Neuropsychiatric correlates of epilepsy

### Diagnosis

Several diagnostic pitfalls should be avoided, but perhaps the most challenging for practitioners in child and adolescent mental health is distinguishing intentional ‘bad behaviour’ from the unusual manifestations associated with frontal lobe seizures. In contrast to many other seizure types, frontal lobe seizures are often brief, repeated and typically nocturnal, with apparently voluntary vocalisation, retention of consciousness and recollection of the episodes after they have occurred. There may be subjective feelings of difficulty breathing; the child may be afraid to go to sleep for fear of having these disturbing nocturnal episodes. Antiseizure medication such as carbamazepine or levetiracetam can be highly effective in controlling these seizures. The simple recommended rule to follow is: if in doubt about the diagnosis of epilepsy, refer to an epilepsy specialist.

### Treatment

The most common treatment pitfall to be avoided by the practitioner managing children with epilepsy is the failure to treat psychiatric disorders adequately because of an inappropriate concern about exacerbating seizures (Besag et al., 2016). Misleading conclusions were drawn from past studies because of a lack of recognition that many psychiatric disorders, including ADHD and depression, are associated with epilepsy, implying seizures in these disorders were often not the result of the psychotropic medication but were, instead, the result of an association with the disorder itself. A good knowledge of valid evidence allows these pitfalls to be avoided. What are the common psychiatric conditions associated with epilepsy and what is the risk of treating them with standard medication? Overall, stimulant medication for ADHD (Cortese et al., 2013), SSRIs for anxiety, depression and obsessive-compulsive disorder (Alper et al., 2007), and melatonin for sleep-onset disorder (Bruni et al., 2015) do not appear in general to exacerbate seizures. The risk of exacerbating seizures with low-dose risperidone when used by some practitioners for anxiety treatment seems to be low, but this compound should only be used after a fair trial of non-pharmacological interventions and SSRIs if symptoms are still impairing. When treating psychosis, the risk of seizure exacerbation is related to the dose of antipsychotic medication. Therefore, practitioners should use a minimal effective dose. Notably, the distress caused by the psychosis might be far more than the distress caused by a seizure exacerbation.

Although the risk of seizure exacerbation with most psychotropic medications is generally small, certain medications probably should be avoided, if possible, including bupropion, imipramine, clomipramine, alprazolam, chlorpromazine, quetiapine, olanzapine and clozapine because there is evidence for seizure precipitation/exacerbation (Alper et al., 2007). However, even with many of these medications, including clozapine, if the dose is kept low, the risk is probably small. Knowledge of which medications should not be used or, if they are prescribed, should be monitored closely, allows the clinician to treat the individual with confidence and to avoid the pitfall of failing to treat psychiatric conditions because of inappropriate fear of seizure exacerbation.

## Psychosis

### Accurate differential diagnosis

Psychotic disorders are infrequent in children, with increasing prevalence in adolescence. However, children and adolescents report psychotic-like experiences and psychotic symptoms far more frequently, more likely due to mental health problems other than psychosis (Sunshine and McClellan, 2023). The diagnosis of a psychotic disorder in children and adolescents should involve a thorough process, based not only on the report of hallucinations and other subjective experiences but also including at least a complete psychopathological assessment, description of behavioural manifestations, attention to context and reports of informants other than the patient, as well as medical clearance. Non-specific reports of psychotic-like experiences are more generally associated with trauma and features of borderline personality disorder (as discussed in Part I paper, the diagnosis of personality disorder in adolescence is controversial, with some practitioners/researchers endorsing this diagnosis, and others using the term *emerging personality disorder* (Elvins and Kaess, 2022)). Negative symptoms and thought disorders present in schizophrenia may be difficult to differentiate from developmental disorders, so documenting deviation from baseline functioning and the presence of positive symptoms is key (Sunshine and McClellan 2023).

### Addressing treatment-related adverse events

Compared to adults, children and adolescents seem more susceptible to adverse events associated with antipsychotics such as acute extrapyramidal side effects, sedation, hyperprolactinemia, weight gain, and metabolic abnormalities (Correll et al., 2022). This, together with minimum differences in efficacy between antipsychotics other than clozapine (Pagsberg et al. 2017), advises to select antipsychotics by safety profile and to monitor regularly tolerability, weight, vital signs and relevant laboratory results during their use. Adverse events monitoring may be challenging, particularly in ambulatory settings and in patients in need of long-term treatments. Adverse events such as tardive dyskinesia, which may also happen in children and adolescents with antipsychotic treatment, are frequently overlooked. Sedation is another frequent side effect that impairs social life and school performance in children and adolescents. Sedation should not be mistaken for cognitive impairment and should be managed by choosing less sedating agents and prescribing most of the daily dose at bedtime (Sunshine and McClellan 2023).

### Dosing

When using antipsychotics in young people, particularly in children, to adjust doses it is important to consider efficacy and tolerability, given that adjusting dosages based solely on body weight has not been found adequate (Liang et al., 2023). Although a general recommendation for antipsychotic therapy is to start slow and go slow, one common pitfall is not to use full dosages due to concerns about avoiding adverse events. In the event of a lack of efficacy, clinicians should raise dosages to the maximum recommended while monitoring for adverse events. However, increasing antipsychotic dosages above the maximum recommended dose, a common pitfall in those cases with inadequate response, should also be avoided, as it is associated with greater differences in side effects than in efficacy (Correll et al. 2022). It is important to note that abrupt cessation of antipsychotic medications can also cause withdrawal dyskinesia and a syndrome of deteriorating behaviour in children and adolescents (Sunshine and McClellan 2023).

### Management of treatment-resistant psychosis

Management of treatment resistance is also challenging in children and adolescents with psychosis. There is high variability, across practitioners, in the waiting time before switching to another antipsychotic in case of treatment non-response (Correll et al. 2022). Although recommendations from guidelines may differ, waiting more than 6 weeks in case of no improvement in psychotic symptoms or functioning is not supported by evidence and should be avoided (Correll et al. 2022). A common practice in case of non-response, besides the increase in dosages above the maximum recommended dose, is to use an antipsychotic combination. However, this practice is not supported by evidence and may lead to more adverse events (Correll et al. 2022). Clozapine, the only antipsychotic treatment with demonstrated superiority in efficacy in children and adolescents with schizophrenia (Pagsberg et al., 2017), is still underused but should be considered in treatment-resistant schizophrenia after two adequate but unsuccessful monotherapy trials.

### Duration of the antipsychotic treatments in youth who recovered after the first episode of psychosis

There is currently no established consensus regarding the duration of the antipsychotic treatments in youth who recovered after the first episode of psychosis. Duration of treatment needs to be individualised, but the most common recommendation in clinical guidelines of early psychosis is to continue treatment with antipsychotics at least for 12 months after the first episode (https://www.orygen.org.au/Campus/Expert-Network/Resources/Free/Clinical-Practice/Australian-Clinical-Guidelines-for-Early-Psychosis/Australian-Clinical-Guidelines-for-Early-Psychosis.aspx). The process of decision-making regarding continuation or withdrawal of medication needs to be individualised and developed together with the patient and their family, and should include consideration of treatment-related (such as initial response or adverse events) and disease-related factors (diagnosis, premorbid adjustment, duration of untreated psychosis, acute or slow onset among others). In case of medication withdrawal, dose reduction may be undertaken gradually, over a number of months, with regular monitoring of potential signs and symptoms of relapse.

## Conclusion

As highlighted in a recent Position Paper (Cortese et al., 2024), there are many unmet needs but also opportunities, alongside possible risks to consider, regarding the pharmacological treatment of mental health conditions in children and adolescents. Addressing the pitfalls in the clinical practice with the psychopharmacological treatment of children and adolescents is key to maximise the benefits of psychopharmacotherapy. While we have endeavoured here to rely on available evidence, integrating it with our own clinical experience, we look forward to additional high-level empirical evidence which will inform future clinical practice and precision medicine approaches in the field (Cortese, 2021).
